# Angiopoietin-1 deficiency increases renal capillary rarefaction and tubulointerstitial fibrosis in mice

**DOI:** 10.1371/journal.pone.0189433

**Published:** 2018-01-02

**Authors:** Krishnapriya Loganathan, Ebtisam Salem Said, Emily Winterrowd, Martina Orebrand, Liqun He, Michael Vanlandewijck, Christer Betsholtz, Susan E. Quaggin, Marie Jeansson

**Affiliations:** 1 Department of Immunology, Genetics and Pathology, Uppsala University, Uppsala, Sweden; 2 Department of Neurosurgery, Tianjin Medical University General Hospital, Tianjin Neurological Institute, Key Laboratory of Post-Neuroinjury Neuro-Repair and Regeneration in Central Nervous System, Ministry of Education and Tianjin City, Tianjin, China; 3 Integrated Cardio Metabolic Centre, Karolinska Institutet, Huddinge, Sweden; 4 Feinberg Cardiovascular Research Institute, Northwestern University, Chicago, IL, United States of America; 5 Division of Nephrology and Hypertension, Northwestern University, Chicago, IL, United States of America; UCL Institute of Child Health, UNITED KINGDOM

## Abstract

Presence of tubulointerstitial fibrosis is predictive of progressive decline in kidney function, independent of its underlying cause. Injury to the renal microvasculature is a major factor in the progression of fibrosis and identification of factors that regulate endothelium in fibrosis is desirable as they might be candidate targets for treatment of kidney diseases. The current study investigates how loss of Angipoietin-1 (Angpt1), a ligand for endothelial tyrosine-kinase receptor Tek (also called Tie2), affects tubulointerstitial fibrosis and renal microvasculature. Inducible Angpt1 knockout mice were subjected to unilateral ureteral obstruction (UUO) to induce fibrosis, and kidneys were collected at different time points up to 10 days after obstruction. Staining for aSMA showed that Angpt1 deficient kidneys had significantly more fibrosis compared to wildtype mice 3, 6, and 10 days after UUO. Further investigation 3 days after UUO showed a significant increase of Col1a1 and vimentin in Angpt1 deficient mice, as well as increased gene expression of *Tgfb1*, *Col1a1*, *Fn1*, and *CD44*. Kidney injury molecule 1 (*Kim1/Havcr1*) was significantly more increased in Angpt1 deficient mice 1 and 3 days after UUO, suggesting a more severe injury early in the fibrotic process in Angpt1 deficient mice. Staining for endomucin showed that capillary rarefaction was evident 3 days after UUO and Angpt1 deficient mice had significantly less capillaries 6 and 10 days after UUO compared to UUO kidneys in wildtype mice. RNA sequencing revealed downregulation of several markers for endothelial cells 3 days after UUO, and that Angpt1 deficient mice had a further downregulation of *Emcn*, *Plvap*, *Pecam1*, *Erg*, and *Tek*. Our results suggest that loss of Angpt1 is central in capillary rarefaction and fibrogenesis and propose that manipulations to maintain Angpt1 levels may slow down fibrosis progression.

## Introduction

Renal fibrosis, characterized by interstitial myofibroblasts and excessive extracellular matrix deposition, is the final common pathway of chronic kidney disease (CKD) [1–3]. Myofibroblasts are the culprit of renal fibrosis and their cellular origin is an open field of investigation and has been debated [3–7]. Mounting evidence has demonstrated that loss of renal peritubular capillaries correlates with severity of fibrosis in both patients and rodent models of CKD, reviewed in [8]. There is currently no effective treatment for fibrosis and identification of factors that regulate fibrotic responses or have endothelial cell protective effects is highly warranted, as these will be new candidate therapeutic targets.

Angiopoietin-1 (Angpt1) is a vascular growth factor that binds to tyrosine kinase receptor Tek, mainly expressed on endothelial cells [9, 10]. Angpt1 induced Tek activation results in endothelial cell survival and quiescence. Angiopoietin-2 (Angpt2) is endothelial derived and in most cases functions as an antagonist on Tek signaling in blood vessels and most likely as an agonist in lymphatic endothelium [11–13]. Angpt2 upregulation results in increased permeability and destabilization of vasculature, priming the endothelium for inflammation and angiogenesis.

Angpt1 is important during vascular development and required in settings of vascular stress, but appears to be dispensable in mature quiescent vessels [9, 14]. In the last few years, several publications suggest involvement of the Angpt/Tek system in several diseases with endothelial dysfunction including diabetes, sepsis, cancer, malaria and fibrosis, and increased ANGPT2/ANGPT1 levels appear to be associated with adverse outcomes [14–24]. Similarly, patients with CKD have been shown to have decreased levels of ANGPT1 and increased levels of ANGPT2 [25].

We have previously demonstrated that loss of Angpt1 predisposes to an increased fibrotic response in wound-healing and in experimental diabetes [14], leading us to hypothesize that Angpt1 has a role in fibrogenesis. Previous studies examining the therapeutic role of Angpt1 in kidney disease have shown promising results. COMP-Angpt1 (cartilage oligomeric matrix protein-Angpt1) is a more soluble and potent Tek activator than naive Angpt1. Gene delivery of COMP-Angpt1 in mice subjected to unilateral ureter obstruction (UUO), cyclosporine induced injury or ischemia-reperfusion injury attenuated tubular injury and tubulointerstitial fibrosis while protecting peritubular capillaries [26–28]. Furthermore, COMP-Angpt1 and podocyte-specific overexpression of Angpt1 had renoprotective roles in diabetes [29, 30]. More recently, Singh *et al* showed that conditional overexpression of Angpt1 in tubular cells attenuated renal fibrosis after UUO [31]. In contrast, Angpt1 treatment was associated with increased fibrosis in the folic acid induced model of fibrogenesis [32]. Based on our previous data together with published studies, we hypothesize that loss of Angpt1 would increase renal tubulointerstitial fibrosis.

## Materials and methods

### Mice

All experiments were approved by the Animal Care Committee of Mount Sinai Hospital (Toronto, ON, Canada) and by the Uppsala Committee of Ethics of Animal Experiments (permit numbers C122412/13 and C110/13) and were conducted according to guidelines established by the Canadian Council on Animal Care and the Swedish Board of Agriculture.

Floxed *Angpt1* mice crossed to the ROSA-rtTA and tetO-Cre transgenic system was used to generate inducible whole body knockout of Angpt1 as described previously [14]. In short, knockout was induced at embryonic day 16.5 by administration of doxycycline in the pregnant dam’s drinking water until weaning. To improve knockout of Angpt1, the breeding female had heterozygous germline deletion of Angpt1 (Angpt1 w/del, ROSA-rtTA, tetO-Cre). Controls (WT) were Angpt1 w/w littermates with ROSA-rtTA and tetO-Cre. Both Angpt1KO and WT mice received doxycycline. Mice were on a mixed background. Both female and male mice were used in all experiments as the UUO model has not shown any gender differences [33]. A whole body knockout model was used as the expression of Angpt1 has been suggested to occur from several cell types. The early induction at embryonic day 16.5 gives a good excision of the gene without any known renal phenotype [14]. We know from experience that later induction (after birth) reduces excision efficacy.

Mice were genotyped by PCR using the following primer pairs; Angpt1 flox (for 5’-CAATGCCAGAGGTTCTTGTGAA and rev 5’-TCAAAGCAACATATCATGTGCA, wt 233 bp, flox 328 bp), Angpt1 del (for 5’-CAATGCCAGAGGTTCTTGTGAA and rev 5’-TGTGAGCAAAACCCCTTTC, 481 bp), and ROSA-rtTA (for 5’-GAGTTCTCTGCTGCCTCCTG and rev 5’-AGCTCTAATGCGCTGTTAAT), general Cre allele (for 5’-ATGTCCAATTTACTGACCG and rev 5’-CGCCGCATAACCAAGTGAA, 673 bp).

### Unilateral ureteral obstruction (UUO)

Fibrosis was induced by unilateral ureteral obstruction [34]. Adult mice (8–12 weeks) were used for all experiments. Anesthesia was induced and maintained by inhalation of isoflurane (~2% vol/vol) mixed with air (~0.5 l/min). Analgesia (Karprofen 5 mg/kg s.c., Norocarp, N-vet AB, Sweden) was administered before surgery and also daily two days post surgery. Clippers were used to shave the area of the incision (midline of abdomen). The incision area was swiped with 70% alcohol and iodine solution. An incision was made just left of the midline and the left ureter was exposed and ligated at 2 places using 4–0 silk. The peritoneum and skin were sutured with 6–0 silk. Mice were euthanized 3.5 hours, 1, 3, 6, and 10 days after UUO, at which time both CL (contralateral) and UUO (obstructed) kidneys were dissected. Kidneys were divided and fixed in 4% paraformaldehyde for 4 hours or snap frozen and stored at -80°C. Fixed kidneys were paraffin embedded and sectioned.

### Antibodies and reagents

Commercially available antibodies were obtained as follows: mouse anti-mouse α-smooth muscle actin (aSMA)-Cy3 (C6198, Sigma), rat anti-mouse endomucin (Ab106100, Abcam), rabbit anti-mouse Ki67 (ab15580, Abcam), rabbit anti-vimentin (Ab92547, Abcam), secondary antibodies from Thermo Fisher Scientific, mouse anti-mouse GAPDH-HRP (ab9482, Abcam), and donkey anti-rabbit-HRP (NA934, GE healthcare Life Sciences). Nuclei were stained with Hoechst 33342 (Thermo Fisher Scientific). Apoptotic cells were labelled with Click-It Plus TUNEL assay (C10619, Thermo Fisher Scientific).

### Image analysis

Immunohistochemistry was performed on 5 μm thick paraffin sections after rehydration and heat mediated antigen retrieval at pH 9. Kidney cortex was imaged (minimum 10 images/mouse) at 400x using a Leica SP8 confocal microscope. For estimation of fibrosis, aSMA positive staining was quantified in each image using the default threshold setting utilizing ImageJ (NIH). Large arteries and glomeruli were excluded. Fibrotic area was expressed a percentage of the whole image area. Average aSMA areas from individual mice were used to calculate the average for group comparisons. Vascular area was calculated using endomucin and Hoechst staining of sections. Quantification of vascular area was done in the same way as for aSMA area above and correlated to the number of nuclei per image, counted in CellProfiler [35]. Apoptotic cells (TUNEL staining) and proliferating cells (Ki67 staining) were counted in 5 image fields /mouse and correlated to the total number of nuclei. Image acquisition and analysis were performed blinded.

### Quantitative real-time PCR

Trizol (Invitrogen) was used to extract mRNA according to the manufacturer’s protocol, followed by cDNA synthesis using iScript reverse transcription supermix (BioRad). Real time PCR was performed using 20 ng of cDNA with iTaq universal SYBR Green supermix (BioRad) and appropriate primers on a CFX-96 Real Time system (BioRad). Expression results were normalized to endogenous control *Hprt* and relative quantification was done using the Livak method (2^-ΔΔCT^) [36]. The following primer pairs were used for analysis; *Hprt* (for 5’- GGCTATAAGTTCTTTGCTGACCTG and rev 5’-AACTTTTATGTCCCCCGTTGA), *Angpt1* (for 5’-GGGGGAGGTTGGACAGTAA and rev 5’-CATCAGCTCAATCCTCAGC), *Fn1* (for 5’-AGATTGGCGACAAGTGGAGG and rev 5’-GGTAGGGCTTTTCCCAGGTC), *Havcr1/Kim1* (for 5’-TGGAGATTCCTGGATGGT and rev 5’-GAGGTAGAGACTCTGGTTGA), *Angpt2* (for 5’-GATCTTCCTCCAGCCCCTAC and rev 5’-TTTGTGCTGCTGTCTGGTTC), *Tek* (for 5’-TGGAGTCAGCTTGCTCCTTT and rev 5’-ACCTCCAGTGGATCTTGGTG), *Vegfa* (for 5’-GGTTCCAGAAGGGAGAGGAG and rev 5’-GCACCCAAGAGAGCAGAAAG), *Pecam1* (for 5’-TTGAGCCTCACCAAGAGAACGGAA and rev 5’-AATCCAGGAATCGGCTGCTCTTCT), *Icam1* (for 5’-CAGTCCGCTGTGCTTTGAGA and rev 5’-CAGAGGTCTCAGCTCCACAC), and *Adgre1 (F4/80)* (for 5’-ACAGTACGATGTGGGGCTTT and rev 5’-GTGTGGTCATCCCCCATCTG). In addition, the following probes with Taqman gene expression master mix (Applied Biosystems) were used for expression analysis: *Hprt* (Mm03024075_m1), *Col1a1* (Mm00801666_g1) and *CD44* (Mm01277161_m1). Data from UUO kidneys were correlated to corresponding CL kidney to reduce variability between mice. After calculating average for each group they were expressed as fold change compared to WT CL kidney.

### RNA sequencing data

RNA sequencing (RNA-seq) was performed on kidneys at baseline (only doxycycline) from WT and Angpt1KO mice and 3 days after UUO, WT and Angpt1KO (n = 3/group). Total mRNA was isolated using Rneasy Mini kit according to the manufacturer’s instructions (Qiagen). A cDNA library was made using SMARTer Stranded Total RNA Sample Prep Kit (Clontech). Sequencing was performed on an Illumina HiSeq 2500. Sequencing data were processed and analyzed using the same method as we previously described [37]. The whole RNA-seq is attached as S1 Dataset.

### Protein analysis

Total protein from kidneys was extracted by homogenizing tissue in RIPA buffer (Thermo Fisher Scientific) containing protease and phosphatase inhibitors (PhosSTOP and Complete, Roche). Following incubation at 4°C and centrifugation, supernatant was collected and measured for protein concentration using a BCA assay (Pierce), aliquoted and stored at -80°C. Protein lysates (10 μg) were used to measure concentrations of pro Collagen, type 1 alpha 1 (Col1a1), utilizing an ELISA kit (ab210579, Abcam) according to the manufacturer’s instructions. Data from UUO kidneys were correlated to corresponding CL kidney to reduce variability between mice. Group average was calculated and expressed as fold change compared to WT CL kidney. For Western blot analysis, 20 μg denatured proteins were separated on 4–20% MiniProtean gels (BioRad) and then transferred to 0.2 μm PVDF membranes. Blots were blocked with 5% BSA and incubated overnight with primary antibody. After washing and incubation with the appropriate HRP-conjugated secondary antibody, proteins were visualized using ECLplus detection reagents (GE, Uppsala, Sweden). To ensure equal protein loading, the same blot was stripped with stripping buffer (L7710A, Interchim) and then incubated with an anti-GAPDH antibody.

### Statistical analysis

Data are expressed as mean ± SEM. Statistical analysis was performed using 2-tailed Student’s t test or one way ANOVA with Bonferroni’s multiple comparisons were appropriate to analyze statistically significant differences between groups. All data was tested for normal distribution and in the case of a skewed distribution logarithmic values were used. RNA-seq data was subjected to a FDR of 5%. A p<0.05 is considered statistically significant.

## Results

### Angpt1 was regulated in renal fibrogenesis

**Fig 1:**
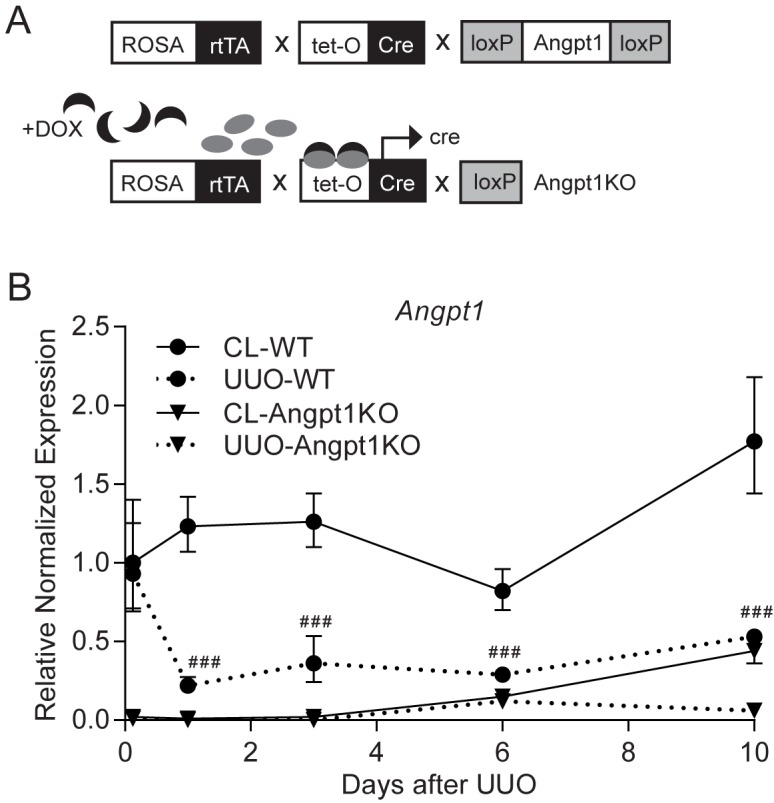
Angpt1 expression was downregulated in UUO kidneys. (A) Schematic diagram of the inducible whole body knock out system to generate Angpt1 knockout mice. (B) Gene expression analysis of *Angpt1* at different time-points (3.5h, 1, 3, 6, and 10 days) after UUO showed a significant down-regulation that started 1 day after UUO in WT mice. Data are expressed as mean ± SEM. Gene expression data in this figure are normalized to *Hprt* and referenced to WT CL kidney 3.5h after UUO (n = 5–6 for 3.5 h, n = 5 for 1 day, n = 13 for 3 day, n = 3–4 for 6 day, and n = 3–5 for 10 day post UUO). ### p<0.001 compared to WT CL kidney 3.5h after UUO.

To study the fibrotic response in mice deficient for Angpt1 we utilized an inducible whole body knock out system activated by doxycycline (DOX) (Fig 1A) [14]. As expected, Angpt1KO mice had very low expression of *Angpt1*, and the excision efficiency was >95% for mice used in the study. As a renal fibrosis model we utilized a murine model of obstruction-induced injury, unilateral ureteral obstruction (UUO). UUO was induced by ligation of the ureter and resulted in the development of fibrotic changes in the interstitium that were characterized by tubular dilatation and interstitial extracellular matrix accumulation as described previously [34]. Measurements of *Angpt1* expression at different time points after UUO showed that *Angpt1* was significantly downregulated in WT mice from day 1 and onward after UUO (Fig 1B), in agreement with a previous study [26].

### Angpt1 deficiency resulted in enhanced renal fibrosis

**Fig 2:**
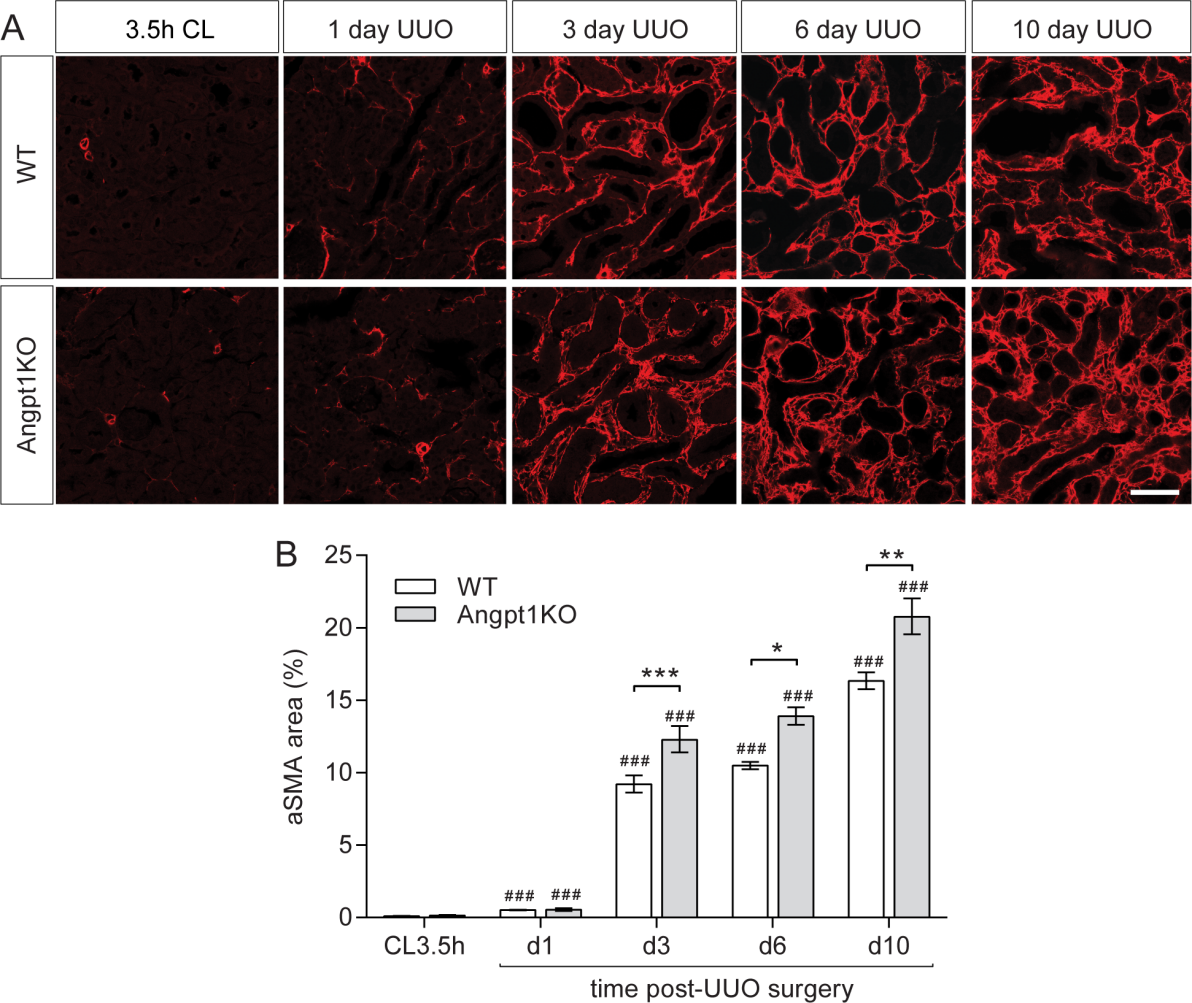
Angpt1 deficiency resulted in increased aSMA area at several time points. (A) UUO in WT and Angpt1KO mice induced tubulointerstitial fibrosis starting 1 day post UUO as seen from aSMA staining of renal cortex. (B) Quantifications of aSMA-positive areas showed a significant increase in fibrotic area in Angpt1KO mice 3 days after UUO and onward. A minimum of 10 images from renal cortex were used from each mouse (n = 3 for day 1, n = 13 for day 3, n = 3–6 for day 6, and n = 3–4 for day 10 post UUO). Scale bar, 50 μm. Data expressed as mean ± SEM. ### p<0.001 compared to WT CL3.5h, *p<0.05, **p<0.01, ***p0.001 compared to WT at the corresponding time point.

Myofibroblasts are characterized by expression of aSMA and production of extracellular matrix components including type I collagens and fibronectin [38, 39]. Additional markers are the cytoskeletal marker vimentin and stem cell marker CD44 [39]. Fibrosis was estimated in kidney sections from WT and Angpt1KO mice by staining for aSMA. Tubulointerstitial staining for aSMA in cortex was significantly elevated already 1 day after UUO compared to unobstructed kidneys (CL). At 3 days after UUO, aSMA area was markedly increased in the renal cortex and Angpt1KO had significantly (p<0.001) more aSMA positive area in UUO kidneys compared to WT mice. Later time points (day 6 and 10 after UUO) also had significantly increased aSMA area in UUO kidneys from Angpt1KO mice compared to WT mice (Fig 2A and 2B).

**Fig 3:**
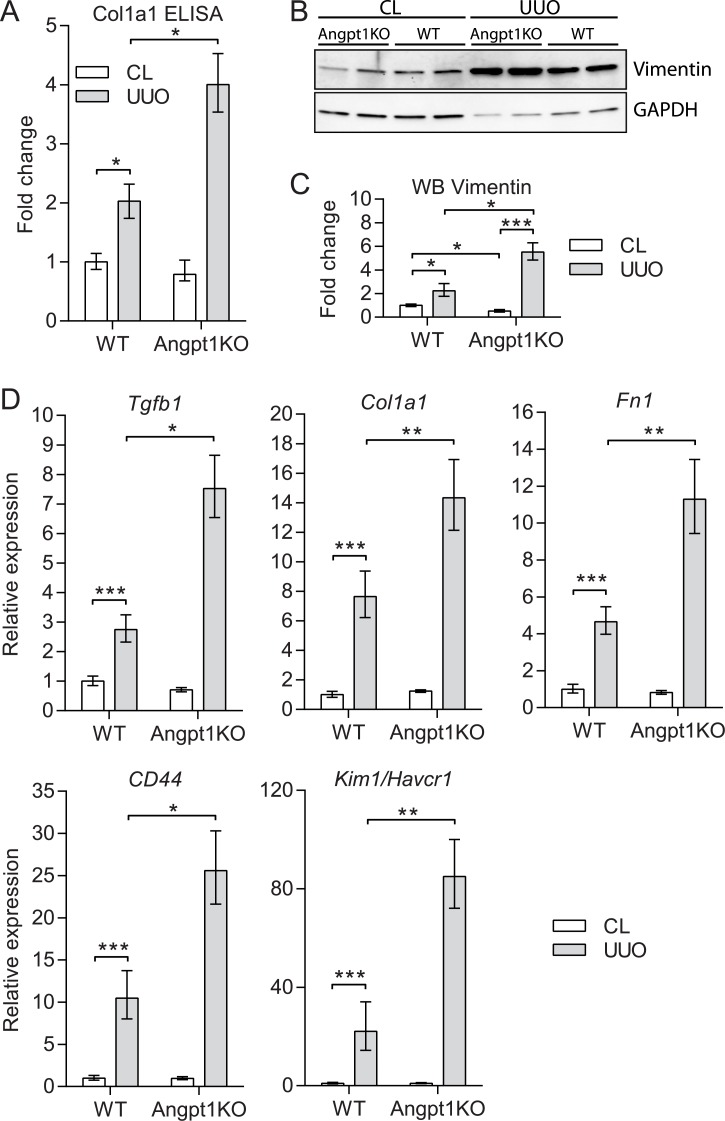
Angpt1 deficiency increased markers for fibrosis and kidney injury after UUO. (A) ELISA showed that Col1a1 protein was significantly increased in Angpt1KO mice 3 days after UUO compared to WT UUO kidneys (n = 13–17). (B) Western blotting showed an increase of Vimentin protein after UUO and a further increase in Angpt1KO 3 days after UUO. (C) Fold change densitometry for Vimentin blots corrected for protein loading (GAPDH) (n = 4). (D) Gene expression analysis showed upregulation of markers for fibrosis and mesenchymal transition (*Tgfb1*, *Col1a1*, *Fn1*, and *CD44*) after UUO and were significantly more increased in Angpt1KO mice 3 days after UUO compared to WT mice (n = 13–17). At the same time point, kidney injury molecule 1 (*Kim1/Havcr1*) was significantly further upregulated in Angpt1KO mice (n = 13–17). Data are expressed as mean ± SEM *p<0.05, **p<0.01, ***p<0.001 as indicated.

To further confirm the increased fibrosis in Angpt1KO mice we performed protein analysis of kidney lysates 3 days after obstruction. ELISA for pro Collagen, type 1 alpha 1, (Col1a1) showed a 2-fold increase of Col1a1 after UUO in WT mice and a significantly higher (4-fold) increase in Angpt1KO mice (Fig 3A). Western blotting for Vimentin, another marker for mesenchymal cells, showed increased protein levels after UUO and was further enhanced in Angpt1KO mice (Fig 3B and 3C, S1 Fig). Vimentin was also expressed by podocytes in the glomerulus. These results demonstrate that Angpt1 deficiency resulted in an enhanced fibrotic response as shown by several upregulated markers of fibrosis.

Transforming growth factor beta (Tgfb) signaling is one of the most studied drivers of fibrosis, and has been shown to induce mesenchymal transition in several cell types. Tgfb1 expression was significantly increased in Angpt1KO mice 3 days after obstruction (Fig 3D). Tgfb1 expression started increasing 1 day after obstruction in both groups (S2A Fig). As expected, *Col1a1*, *Fn1*, and *CD44* were significantly upregulated in WT kidneys after UUO, 8-fold, 4-fold, and 10-fold, respectively, compared to CL kidneys. In Angpt1KO mice these genes were further enhanced by about 2-fold compared to WT UUO kidneys (Fig 3D). *Fn1* and *Col1a1* started increasing in both groups already 1 day after UUO, but did not reach statistical significance (S2B and S2C Fig).

Kidney injury molecule 1 (Kim1/Havcr1) is upregulated in proximal tubule cells after injury but undetectable in healthy kidneys [40, 41]. After injury, it localizes to the apical surface of surviving proximal tubule epithelial cells [42]. In WT kidneys *Kim1* was upregulated 20-fold after UUO, however, in Angpt1KO mice it was upregulated 80-fold after UUO (Fig 3D), suggesting a more severe injury in Angpt1 deficient mice. *Kim1* increased 1 day after UUO and was significantly more upregulated in Angpt1KO mice (S2D Fig).

RNA-seq data for *Col1a1*, *Cd44*, *Fn1*, *Acta2*, *Vim*, *Tgfb1*, and *Kim1* with 3 samples per group showed similar trends but did not always reach statistical significance (S3 Fig).

### Angpt1 deficiency increased capillary rarefaction after UUO

**Fig 4:**
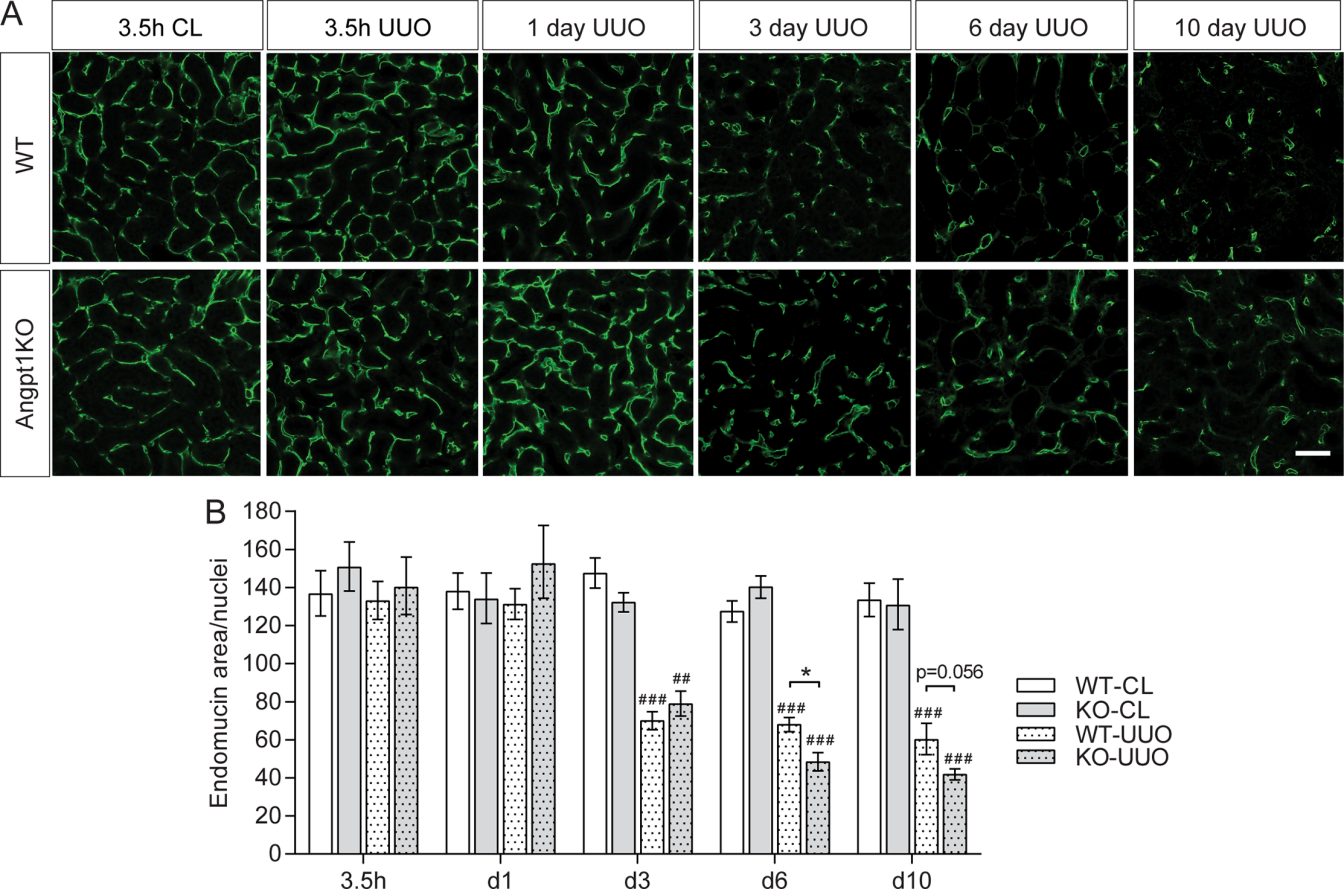
Angpt1 deficiency increased vascular rarefaction after UUO. (A) Vessel area in renal cortex was estimated from endomucin staining correlated to nuclei number at all time points after UUO. (B) Vascular rarefaction started 3 days after UUO and vessel density was significantly lower 3, 6, and 10 days after UUO in both WT and Angpt1KO mice. At 6 and 10 days of UUO, Angpt1KO mice showed a decreased capillary density compared to WT UUO mice. A minimum of 10 images from renal cortex were used from each mouse (n = 4–6 for 3.5h, n = 5–7 for day 1, n = 6–7 for day 3, n = 4–5 for day 6, and n = 4 for day 10 post UUO). Data shown as mean ± SEM. Scale bar, 50 μm. ## p<0.01, ### p<0.001 comparing to WT CL3.5h. *p<0.05 compared to WT at corresponding time point.

Based on several studies of CKD patients and animal models of CKD, it is known that peritubular capillaries disappear in association with progressive interstitial fibrosis [8]. As Angpt1 and Tek are important vascular factors we investigated vessel rarefaction after UUO at different time points. Staining for endomucin, a marker for endothelial cells [43], was used to identify endothelium in renal cortex and correlated to the total number of nuclei in each image. UUO resulted in significant peritubular capillary rarefaction 3, 6 and 10 days after UUO (Fig 4A and 4B). At day 6 and 10, a further reduction of peritubular capillaries was seen in Angpt1KO mice after UUO compared to WT mice (Fig 4A and 4B).

**Fig 5:**
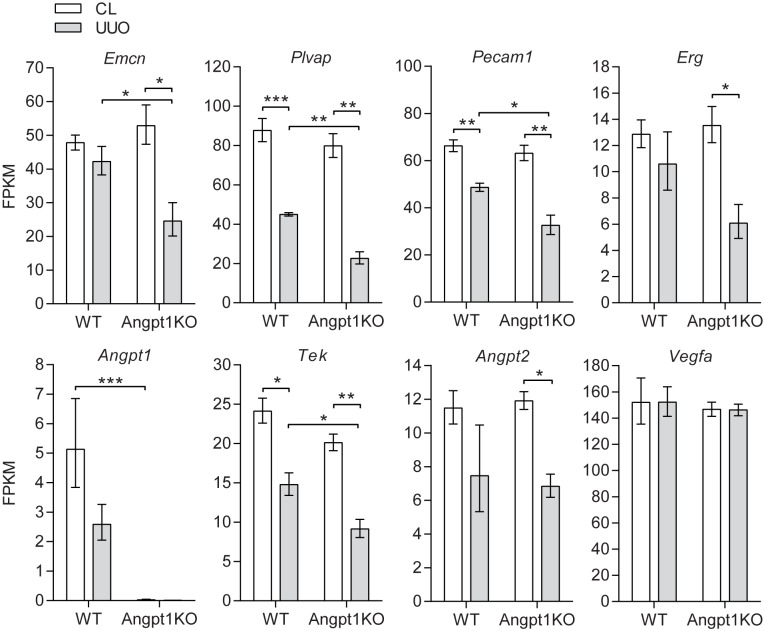
Angpt1 deficiency decreased expression of endothelial markers 3 days after UUO. RNA-seq data at baseline and 3 day UUO kidneys from WT and Angpt1KO mice. Data shown as mean ± SEM. FPKM–Fragments per kilobase million. n = 3 for all groups. *p<0.05, **p<0.01, ***p<0.001 compared to indicated group.

To further investigate regulation of endothelium we performed RNA-seq of kidneys at baseline and 3 days after UUO in both WT and Angpt1KO mice. Sequencing data showed that several markers of capillaries were downregulated after UUO and that some genes were significantly more reduced in Angpt1KO mice, i.e. *Emcn*, *Plvap*, *Pecam*, *Erg*, and *Tek* (Fig 5). Real time PCR showed that *Tek* was significantly downregulated 1 day after UUO in both WT and Angpt1KO to a similar degree, whereas *Pecam1* showed a decreasing trend without reaching statistical significance (S4A and S4B Fig). Sequencing data for *Angpt1* showed the same trend as the result in Fig 1 with *Angpt1* being downregulated after UUO and, as expected, the transcript was lost in Angpt1KO mice (Fig 5). *Angpt2* showed a similar trend as *Tek* and was significantly downregulated in Angpt1KO mice 3 days after UUO but did not reach statistical significance 1 day after UUO which WT mice did (Fig 5, S4C Fig).

**Fig 6:**
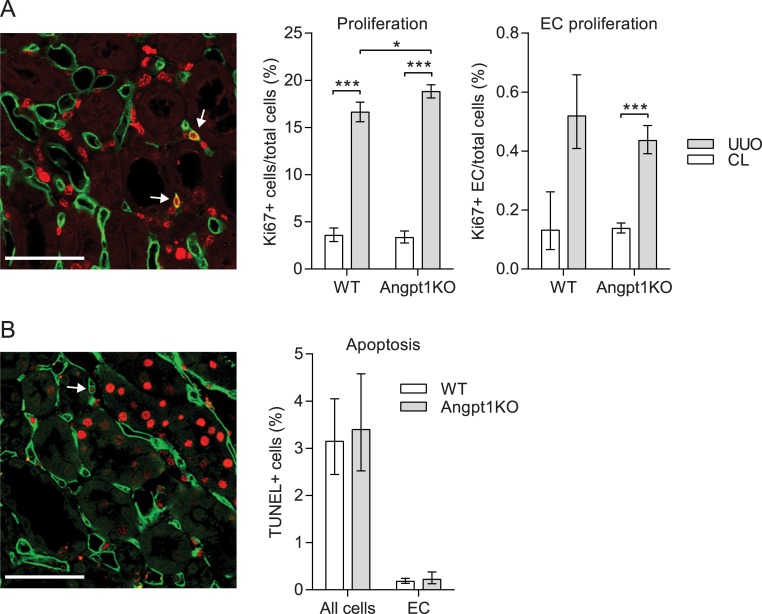
Angpt1 deficiency did not alter endothelial proliferation and apoptosis. (A) Proliferation was estimated from Ki67 staining (red) of renal cortex 3 days after UUO. Endomucin staining (green) and Hoechst (not shown) was used to identify endothelial cells and nuclei, respectively. Nuclei positive for Ki67, surrounded by endomucin was identified as an proliferating endothelial cell (arrow). As expected, proliferation was increased after UUO. Angpt1KO showed a significant increase in overall proliferation compared to WT after UUO; however, there was no difference in endothelial proliferation. (B) Apoptosis was estimated by TUNEL staining (red) of renal cortex 3 days after UUO. Endomucin staining (green) and Hoechst (not shown) was used to identify endothelial cells and nuclei, respectively. Nuclei positive for TUNEL, surrounded by endomucin was identified as an apoptotic endothelial cell (arrow). Scale bar, 50 μm. Data shown as mean ± SEM. n = 3–5 for all groups. *p<0.05, ***p<0.001 compared to indicated group.

The angiogenic factor *Vegfa* was not affected 3 days after UUO but showed a decreasing trend at day 1 in UUO kidneys in both WT and Angpt1KO mice (Fig 5, S4 Fig). Other factors that could affect capillary density are endothelial cell apoptosis and proliferation. Overall proliferation, as measured by Ki67 staining, was increased in UUO kidneys 3 days after obstruction, and Angpt1KO mice had significantly more proliferation (Fig 6A). Angpt1KO mice had significantly more endothelial cell proliferation after UUO compared to CL kidney, however, this did not differ from WT mice. Apoptosis was measured by TUNEL staining 3 days after UUO and was similar in WT and Angpt1KO mice (Fig 6B). Inflammation has also been indicated in fibrogenesis and the Angpt/Tek system is known to be involved in inflammation. In the current study we did not find any difference in inflammatory response to UUO comparing WT and Angpt1 mice for *Adgre1/F480* and *Icam* (S6 Fig).

## Discussion

In the current study we show for the first time that loss of Angpt1 increases tubulointerstitial fibrosis and capillary rarefaction in a murine model of fibrosis, UUO. Angpt1 deficient mice showed an enhanced increase of aSMA area and expression of several markers of fibrosis. Correlated with fibrosis was an increased loss of endothelial markers in Angpt1 deficient mice as seen from immunohistochemistry and RNA-seq experiments. Furthermore, *Kim1/Havcr1*, a marker of proximal tubule damage, was significantly more elevated in Angpt1KO mice before onset of fibrosis, suggesting an important early role for Angpt1 in fibrogenesis.

Previous studies examining the therapeutic role of Angpt1 in kidney disease have shown promising results. Increasing Angpt1 demonstrated beneficial effects in several models of fibrosis including UUO, cyclosporine induced injury, and ischemia-reperfusion injury, with attenuated tubular injury and tubulointerstitial fibrosis while protecting peritubular capillaries [26–28]. COMP-Angpt1 and podocyte-specific overexpression of Angpt1 had renoprotective roles in diabetes [29, 30]. Also, tubular cell specific overexpression of Angpt1 attenuated renal fibrosis after UUO [31]. While several studies have investigated the beneficial effects of Angpt1 therapy, no studies have to our knowledge studied the effects of Angpt1 deficiency in tubulointerstitial fibrosis. In the current study, Angpt1KO resulted in a ~30% increase in tubulointerstitial fibrosis after obstruction compared to WT mice (Fig 2). As there was a marked increase in fibrosis 3 days after UUO, we looked at several markers for fibrosis and mesenchymal cells at this time point, both mRNA and protein level, and found them to be significantly more increased in Angpt1 deficient mice.

Capillary rarefaction is associated with tubulointerstitial fibrosis both in human CKD and animal models of CKD [44]. Furthermore, it has been suggested that interstitial hypoxia due to arteriolar vasoconstriction and/or capillary rarefaction is a primary event in CKD, perhaps both a cause and a result of CKD progression [8]. As Angpt1 and Tek are important vascular factors we investigated vessel rarefaction after UUO at different time points. Endomucin staining showed that UUO resulted in significant peritubular capillary rarefaction at the onset of fibrosis and onward (day 3–10). At day 6 and 10 after UUO peritubular capillaries were significantly more reduced in Angpt1 deficient mice compared to WT mice (Fig 4). In the current study we correlated endomucin staining to the number of nuclei in the same field. When nuclei were counted it was evident that the number of nuclei increased in the UUO kidney from both WT and Angpt1KO 3 days after obstruction and onward (S5 Fig). This effect is probably from a combination of infiltrating cells, proliferation, and constriction of the tissue. Testing showed that the increase in nuclei per field in UUO kidneys could not account for all of the capillary rarefaction seen 3 days after UUO and onward, and more importantly that it could not account for differences between WT and Angpt1KO mice. To further investigate effects on the endothelium we utilized a RNA-seq data set from 3 days after UUO and at baseline from WT and Angpt1KO mice. RNA-seq analysis showed that several genes for endothelium were down regulated after UUO, and that several of them were further downregulated in Angpt1 deficient mice (Fig 5), i.e. *Emcn*, *Plvap*, *Pecam1*, *Erg*, and *Tek*.

Studies with pro-Angpt1 therapies in fibrosis have suggested that preservation of the vasculature is one of Angpt1’s protective mechanisms [26–28, 31], which our results are in line with. However, how Angpt1 is doing this is unclear. Increased loss of endothelial cells in the current study could be due to an increase in apoptosis or loss of proliferation. Counting of apoptotic cells that were positive for endomucin did not reveal any differences between Angpt1 deficient mice and WT mice (Fig 6), however, it should be noted that there were few apoptotic endothelial cells, which could make small differences difficult to detect. Proliferation of endothelial cells and increase in capillary area has been described previously after UUO, preceding capillary rarefaction [45]. In the current study we saw an increase in proliferation after UUO, both overall proliferation and endothelial proliferation. While Angpt1KO mice had an increase in proliferation after UUO compared to WT mice, we could not detect a difference in proliferating endothelial cells (Fig 6). Furthermore, we did not observe any increased staining of capillary area (Fig 4). Why this discrepancy? Lin *et al* showed increased vessel area 2 days after UUO and endothelial cell proliferation peaking 4 days after UUO and still significantly higher when capillary rarefaction occurred 10 days after UUO [45]. In the current study we saw capillary rarefaction already 3 days after UUO, suggesting a faster progression in our model. Mouse strain background is known to affect the progression of kidney disease and while Lin *et al* used mice on a BL6 background the mice in the current study were on a mixed background. Hence, it is possible that an increased endothelial area did not occur in our model or that we missed a time point when it did. Another possibility for a reduction in endothelial markers in our model is endothelial-mesenchymal transition, and that Angpt1-Tek signaling supports endothelial cell identity. Endothelial-mesenchymal transition has been suggested to contribute to a portion of myofibroblasts in models of CKD [6], but more studies are needed to investigate Angpt1-Tek signaling in this setting.

Another interesting finding in the current study is the differential expression of *Kim1*, a marker of tubular injury, between WT and Angpt1KO mice after UUO. *Kim1* was upregulated already 1 day after UUO, and significantly more upregulated in Angpt1 KO kidneys (Fig 3, S2D Fig). There is convincing evidence from both animal models and clinical studies that Kim1 could serve as a biomarker of kidney injury as it is rapidly expressed in proximal tubular epithelial cells after injury [46]. The significant increase of *Kim1* in Angpt1 deficient mice suggests that these mice suffer a more severe injury after UUO than WT controls. Moreover, Kim1 is very much linked to fibrosis as Kim1 overexpression in epithelial cells resulted in progressive renal interstitial inflammation and fibrosis [47]. A potent stimulus for inducing Kim1 is tubular hypoxia [42], thus warranting further studies of vascular function in Angpt1 deficient mice in fibrosis. Kim1 also have important functions in inflammation and its expression results in elevated levels of proinflammatory factors [47]. Angpt1 is known to have anti-inflammatory functions and tubular overexpression of Angpt1 decreased inflammatory markers after UUO [31]. As expected, we could see an increase of inflammatory markers, *Adgre1* (F4/80) and *Icam1* after UUO but no differences between WT and Angpt1 deficient mice (S6 Fig). Inflammation was not studied extensively in the current study as the analgesic/anti-inflammatory drug Karprofen was used, hence blunting the inflammatory response.

Interestingly, measurements of Angpt1 expression at different time points showed that UUO in itself decreased *Angpt1* expression significantly in WT mice from day 1 and onward after UUO (Fig 1), in agreement with a previous study [26]. A complication of the decrease of Angpt1 expression in the current study is that the dose difference between WT kidneys and Angpt1KO kidneys is only about 40%, which may lead to small phenotypic differences that are difficult to detect. On the other hand, this clearly suggests that loss of Angpt1 has an important role early in fibrogenesis. It is clear that in WT kidneys, loss of Angpt1 at day 1 after UUO preceded vessel rarefaction and fibrosis 3 days after UUO. What could be the mechanism for loss of Angpt1? In the kidney, Angpt1 is produced by podocytes and pericytes, and perhaps other cell types. Lineage tracing of pericytes and interstitial fibroblasts show that these cells are a major source of myofibroblasts in fibrogenesis [48]. It is possible that Angpt1 expression would be lost if a cell is activated into a myofibroblast, although this needs to be further investigated. Another explanation could be injury and/or loss of another Angpt1-producing cell during the fibrotic process.

**Fig 7:**
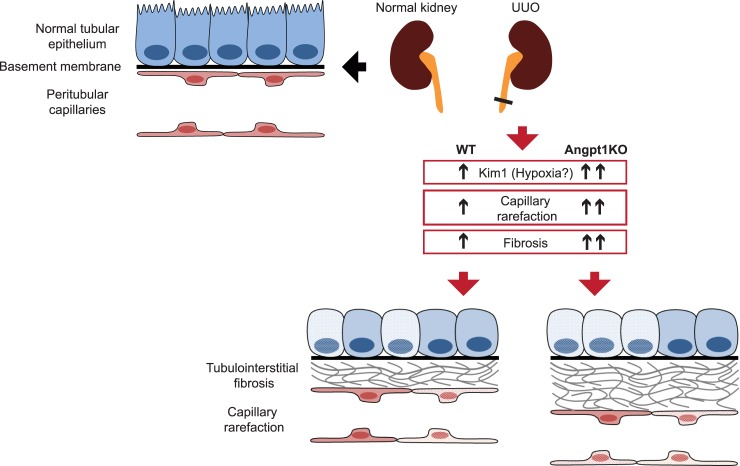
Angpt1 deficiency worsens UUO outcome. A schematic diagram of fibrosis and capillary rarefaction after UUO in Angpt1 deficiency.

In summary, we show that Angpt1 deficiency results in increased fibrosis after UUO, as demonstrated by several markers. Capillary rarefaction occurs at the same time, and is worse in Angpt1 deficient kidneys (Fig 7). Preceding both fibrosis and capillary rarefaction is a drop of *Angpt1* expression in WT kidneys after UUO. At the same time point, kidney injury marker *Kim1* is upregulated and further increased in Angpt1KO kidneys. These data strongly suggest that loss of Angpt1 is a central event early in fibrogenesis and that manipulation to maintain Angpt1 levels may slow down fibrosis progression.

## Supporting information

S1 Dataset RNA-seq data. (XLS)

S1 Fig Western blots. (A) Representative blots for Vimentin and (B) loading control, GAPDH.
(PDF)

S2 Fig Supporting gene expression data—fibrosis. Gene expression for *Tgfb1* (A), *Fn1* (B), Col1a1 (C), and Kim1/Havcr1 (D) 3.5h and 1 day after UUO (n = 5 WT and n = 6 KO for each time point). Data shown as mean ± SEM. ### p<0.001 compared to WT CL kidney at 3.5 h after UUO and *p<0.05 compared to WT CL at corresponding time point.
(PDF)

S3 Fig RNA-seq data—fibrosis. RNA-seq data for genes indicated in fibrosis, mesenchymal transition and kidney injury. n = 3 for all groups. Data shown as mean ± SEM. FPKM–Fragments per kilobase million. *p<0.05 compared to indicated group.
(PDF)

S4 Fig Supporting gene expression data—endothelium. Gene expression for Tek (A), Pecam1 (B), Angpt2 (C), and Vegfa (D) 3.5h and 1 day after UUO (n = 5 WT and n = 6 KO for each time point). Data shown as mean ± SEM. #p<0.05, ### p<0.001 compared to WT CL3.5h kidney.
(PDF)

S5 Fig Nuclei counts. The number of nuclei at different time points for endomucin measurements in Fig 4. A minimum of 10 images from renal cortex were used from each mouse (n = 4–6 for 3.5h, n = 5–7 for day 1, n = 6–7 for day 3, n = 4–5 for day 6, and n = 4 for day 10 post UUO). Data shown as mean ± SEM. **p<0.01, p<0.001 as indicated.
(PDF)

S6 Fig Inflammation. Gene expression level for (A) *Adgre1* (F4/80) and (B) *Icam1* at different time points after UUO (n = 5 WT and n = 6 KO for each time point). (C) RNA-seq data for *Icam1* 3 days after UUO (n = 3 per group). Data shown as mean ± SEM. FPKM–Fragments per kilobase million. #p<0.05, ##p<0.01, ### p<0.001 compared to WT CL3.5h kidney, and *p<0.05, **p<0.01 to indicated group.
(PDF)
