# Supplementary figures and images for: Angiopoietin-1 deficiency increases renal capillary rarefaction and tubulointerstitial fibrosis in mice
Source: PLoS One. 2018 Jan 2;13(1):e0189433. doi: 10.1371/journal.pone.0189433 (PMC5749705; doi:10.1371/journal.pone.0189433)

S1 Fig.

A

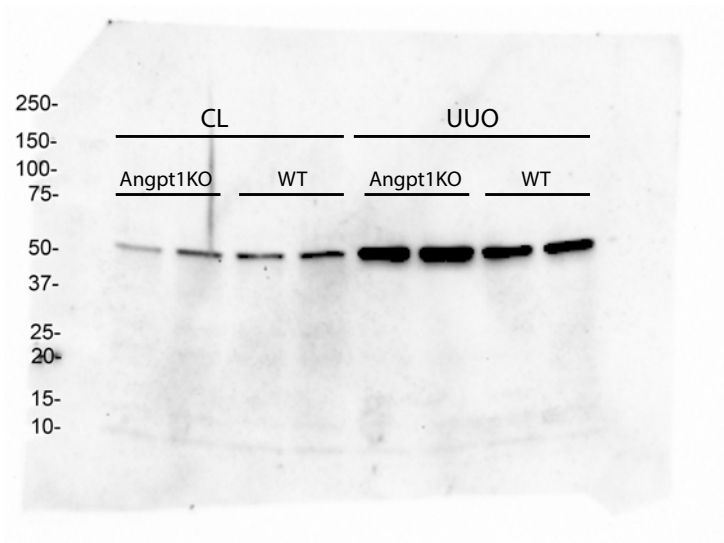

B

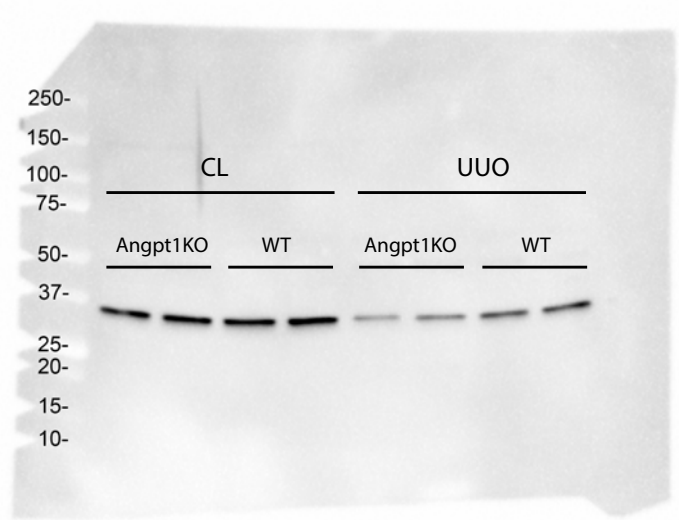

Supplement: S1 Fig — (A) Representative blots for Vimentin and (B) loading control, GAPDH. (PDF) [file pone.0189433.s002.pdf]

S2 Fig.

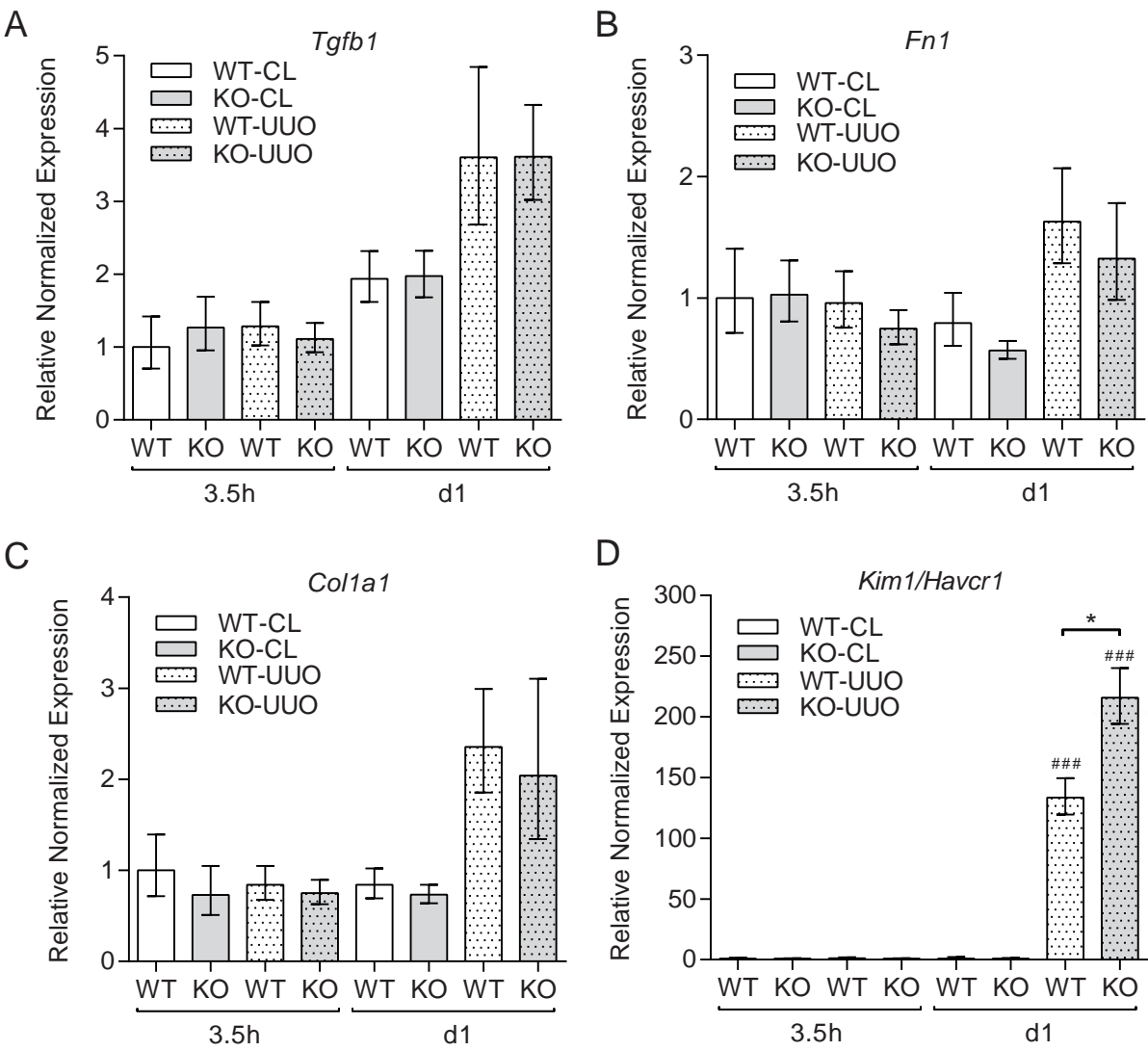

Supplement: S2 Fig — Gene expression for Tgfb1 (A), Fn1 (B), Col1a1 (C), and Kim1/Havcr1 (D) 3.5h and 1 day after UUO (n = 5 WT and n = 6 KO for each time point). Data shown as mean ± SEM. ### p<0.001 compared to WT CL kidney at 3.5 h after UUO and *p<0.05 compared to WT CL at corresponding time point. (PDF) [file pone.0189433.s003.pdf]

S3 Fig.

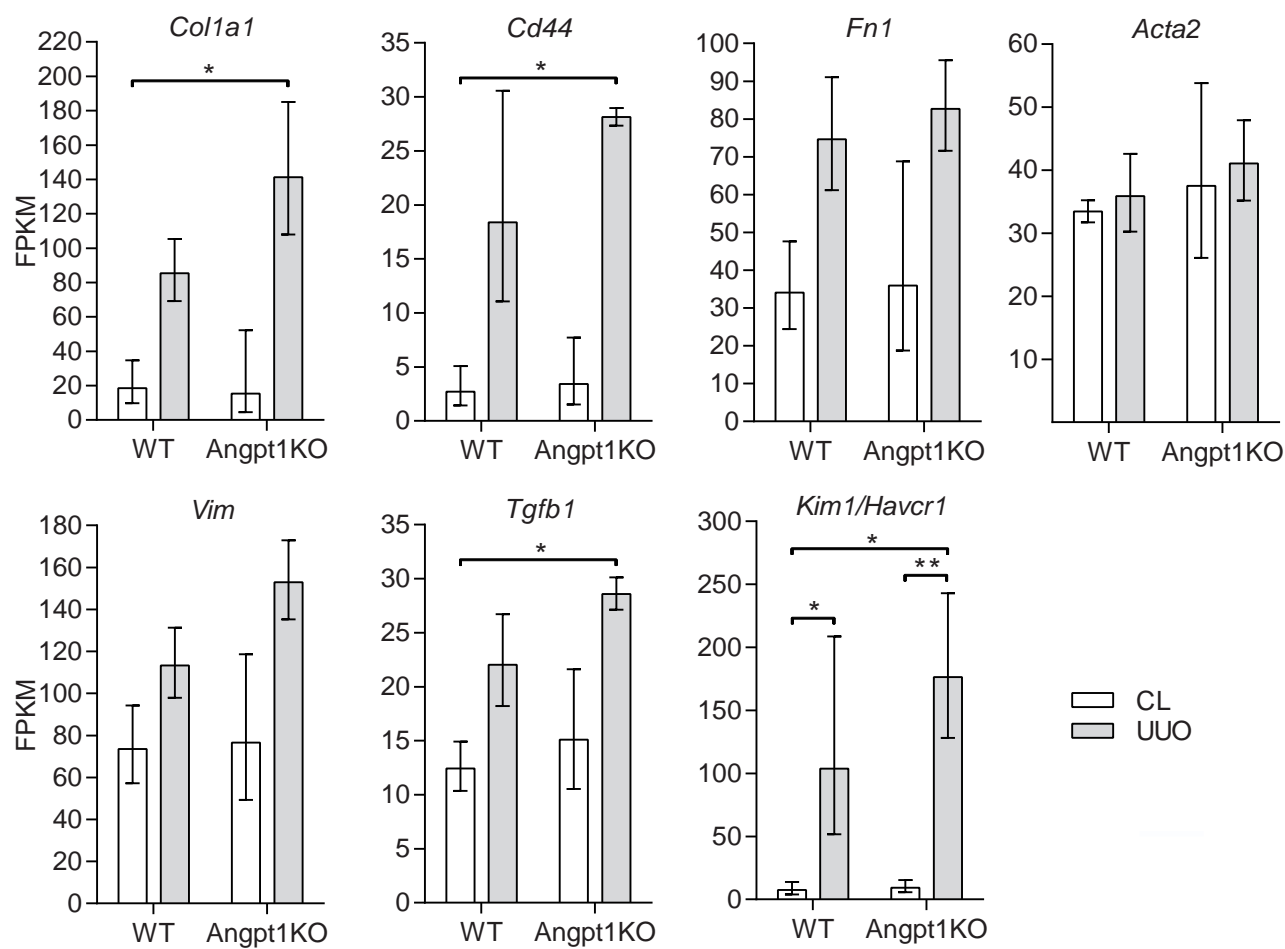

Supplement: S3 Fig — RNA-seq data for genes indicated in fibrosis, mesenchymal transition and kidney injury. n = 3 for all groups. Data shown as mean ± SEM. FPKM–Fragments per kilobase million. *p<0.05 compared to indicated group. (PDF) [file pone.0189433.s004.pdf]

S4 Fig.

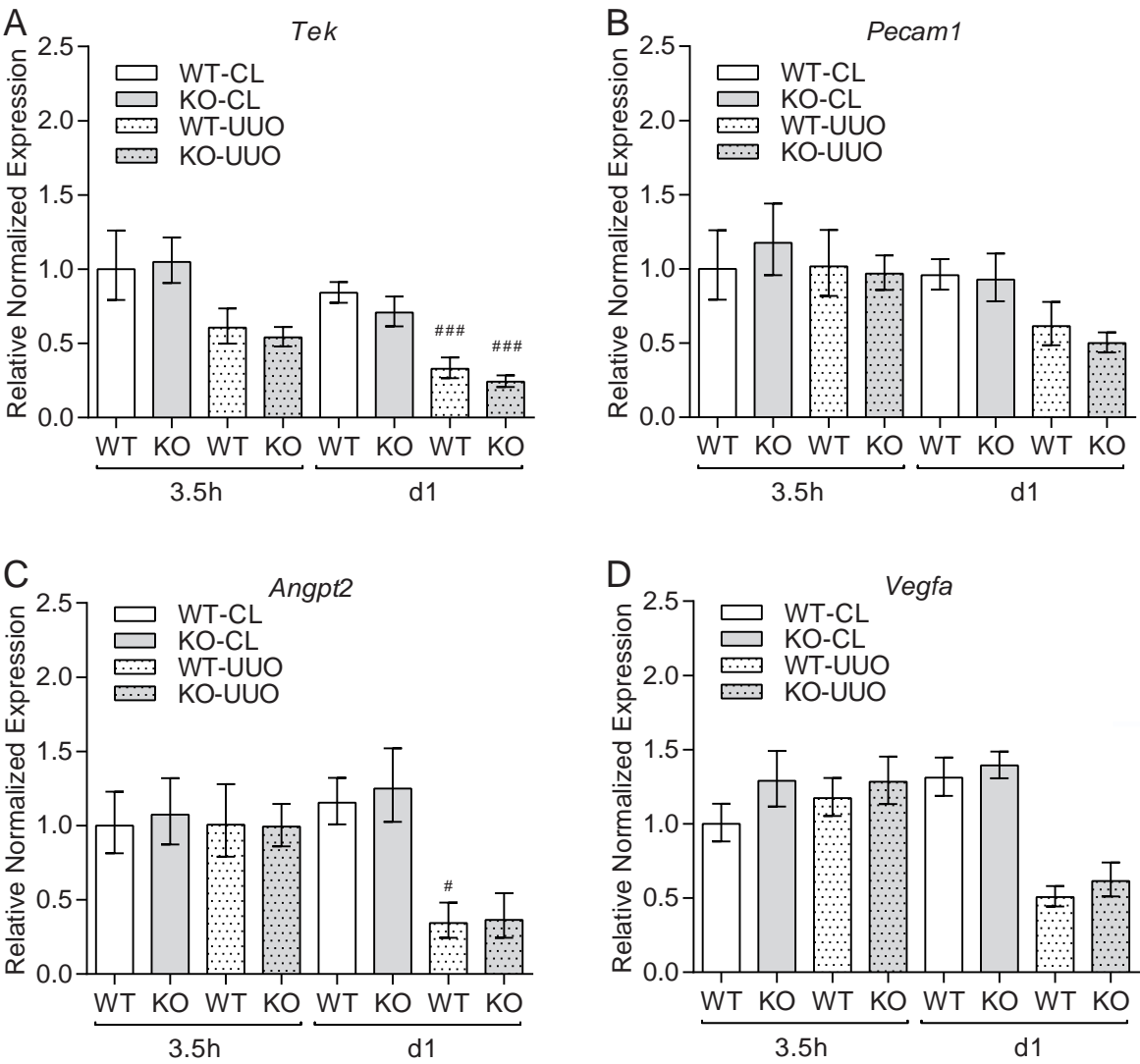

Supplement: S4 Fig — Gene expression for Tek (A), Pecam1 (B), Angpt2 (C), and Vegfa (D) 3.5h and 1 day after UUO (n = 5 WT and n = 6 KO for each time point). Data shown as mean ± SEM. #p<0.05, ### p<0.001 compared to WT CL3.5h kidney. (PDF) [file pone.0189433.s005.pdf]

S5 Fig

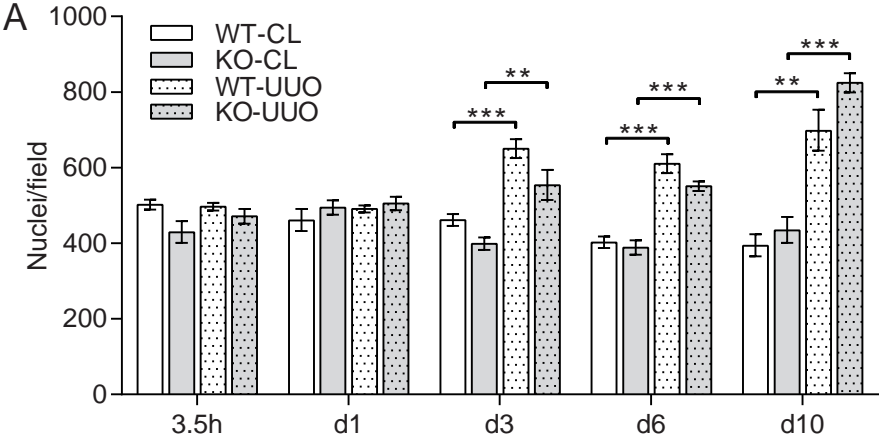

Supplement: S5 Fig — The number of nuclei at different time points for endomucin measurements in Fig 4. A minimum of 10 images from renal cortex were used from each mouse (n = 4–6 for 3.5h, n = 5–7 for day 1, n = 6–7 for day 3, n = 4–5 for day 6, and n = 4 for day 10 post UUO). Data shown as mean ± SEM. **p<0.01, p<0.001 as indicated. (PDF) [file pone.0189433.s006.pdf]

S6 Fig

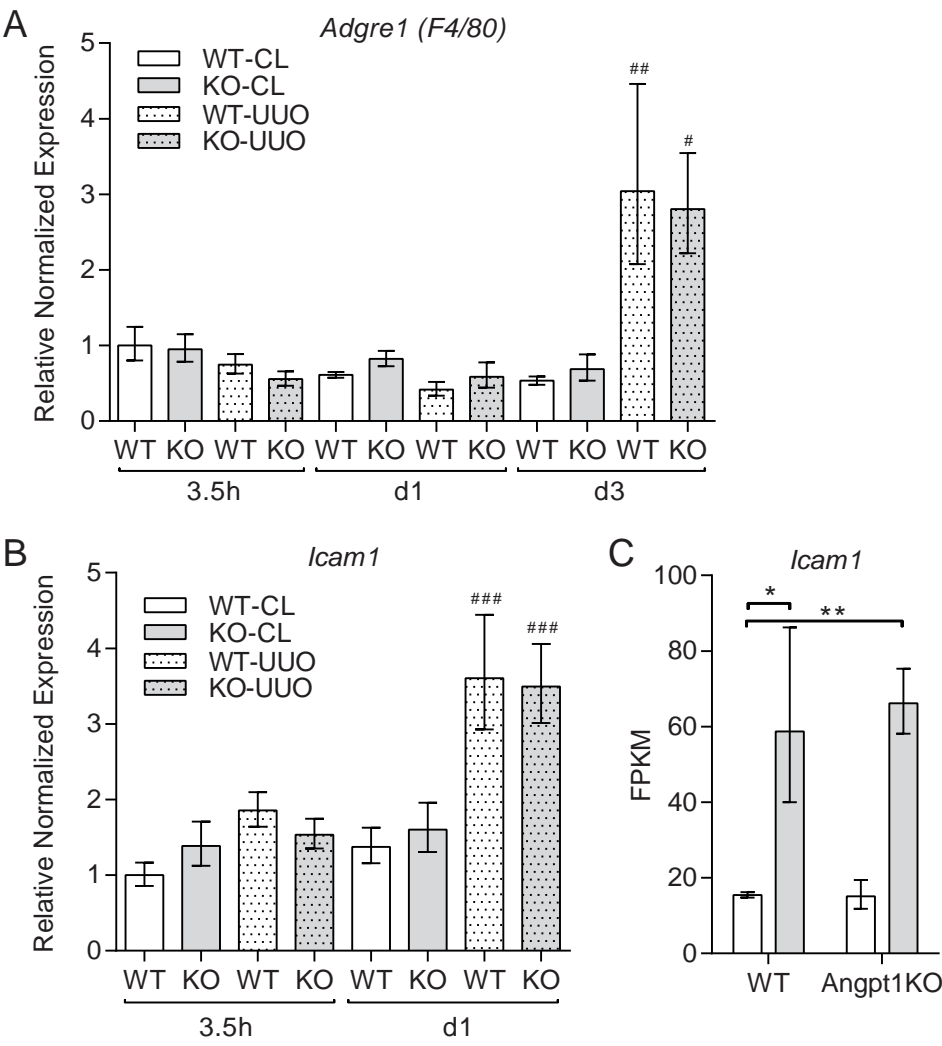

Supplement: S6 Fig — Gene expression level for (A) Adgre1 (F4/80) and (B) Icam1 at different time points after UUO (n = 5 WT and n = 6 KO for each time point). (C) RNA-seq data for Icam1 3 days after UUO (n = 3 per group). Data shown as mean ± SEM. FPKM–Fragments per kilobase million. #p<0.05, ##p<0.01, ### p<0.001 compared to WT CL3.5h kidney, and *p<0.05, **p<0.01 to indicated group. (PDF) [file pone.0189433.s007.pdf]
